# Risk factors and prediction model for delayed bleeding after cold snare polypectomy: a retrospective study

**DOI:** 10.1007/s00384-024-04687-8

**Published:** 2024-07-22

**Authors:** Shuting Wen, Long He, Xiying Zhao, Yingting Li, Xiaofeng Lin, Zhaoli Fu, Wenfang He, Tianwen Liu

**Affiliations:** 1 Department of Gastroenterology, The Second Affiliated Hospital of Guangzhou University of Chinese Medicine, Guangzhou, 510000 China; 2 Department of Digestive Endoscopy, The First Affiliated Hospital of Guangzhou University of Chinese Medicine, Guangzhou, 510000 China

**Keywords:** Colorectal polyp, Cold snare polypectomy, Delayed bleeding, Risk factors, Prediction model

## Abstract

**Background:**

Delayed bleeding (DB) is a serious complication after cold snare polypectomy (CSP) for polyps in the colon. The present study aimed to investigate the incidence and risk factors of DB after CSP and to develop a risk-scoring model for predicting DB.

**Methods:**

A retrospective study was conducted in four Chinese medical institutions. 10650 patients underwent CSP from June 2019 to May 2023. The study analyzed the rate of DB and extracted the general clinical information and polyp-related information of patients with postoperative DB. As a control, non-DB patients who received CSP at the same 4 hospitals were analyzed. A multivariate Cox regression analysis was performed to develop the prediction model. The model was further validated using a Kaplan–Meier log-rank analysis, receiver operating characteristic curve (ROC) plot and risk plot.

**Results:**

In our study, we found a 0.24% rate of DB and the risk factors were history of hypertension, hyperlipidemia, antithrombotics use, antiplatelet use, anticoagulant use, abdominal operation, sigmoid colon lesion, hematoma, cold snare defect protrusion, polyp size, wound size, the grade of wound bleeding, and morphology of Ip. These factors were incorporated into the prediction model for DB after CSP. For 1, 3, and 5 days of bleeding, the AUC of the ROC curve was 0.912, 0.939, and 0.923, respectively. The Kaplan–Meier analysis indicated that the high-risk group had a significantly higher risk of DB than the low-risk group.

**Conclusions:**

This study screened the risk factors and established a prediction model of DB after CSP. The results may help preventing and reducing the DB rate after CSP of colorectal polyps.

**Supplementary information:**

The online version contains supplementary material available at 10.1007/s00384-024-04687-8.

## Introduction

Colorectal cancer (CRC), a prevalent malignancy of the gastrointestinal system, is a major contributor to global cancer-related deaths [1]. It is known that almost 90% of CRC evolves from colorectal adenoma [2]. Therefore, endoscopic resection of colorectal polyps is an effective method for reducing the incidence and mortality of CRC [3]. Recently, cold snare polypectomy (CSP) is commonly used for small colorectal lesions ≤ 1 cm [4]. Compared with traditional hot snare polypectomy, CSP does not require high-frequency electric device and submucosal injection, and has the advantages of convenient operation, low risk of delayed bleeding (DB) and perforation [5]. CSP can also ensure low residual rate and high surgical resection rate [6].

However, some degree of oozing from the wound surface is inevitable after CSP resection [7]. Most of the bleeding can stop by itself within a few seconds to a few minutes, but some lesions will continue to bleed after resection. DB is defined as the occurrence of bleeding within 24 h to several days after CSP. It is often not relevant to mechanical factors after CSP, but rather caused by postoperative coagulopathy, vascular injury, or infection. DB is one of the complications after CSP. Excessive bleeding can lead to serious complications such as shock, which could result in the patient's death if not treated immediately. Therefore, it is important to identify the risk factors for DB after CSP and achieve endoscopic hemostasis immediately, which can help reduce the complications and further improve the safety and efficiency of CSP.

DB has been linked to antiplatelet and anticoagulant use, polyp size larger than 5 mm and rectal lesion [8]. To the best of our knowledge, currently, there have been no relevant research on the prediction model of DB after CSP. In this study, we analyzed the risk factors affecting DB after CSP, and constructed a prediction model, in order to provide a basis for preventing and reducing the DB rate after CSP of colorectal polyps.

## Materials and methods

### Study design and participants

**Fig. 1:**
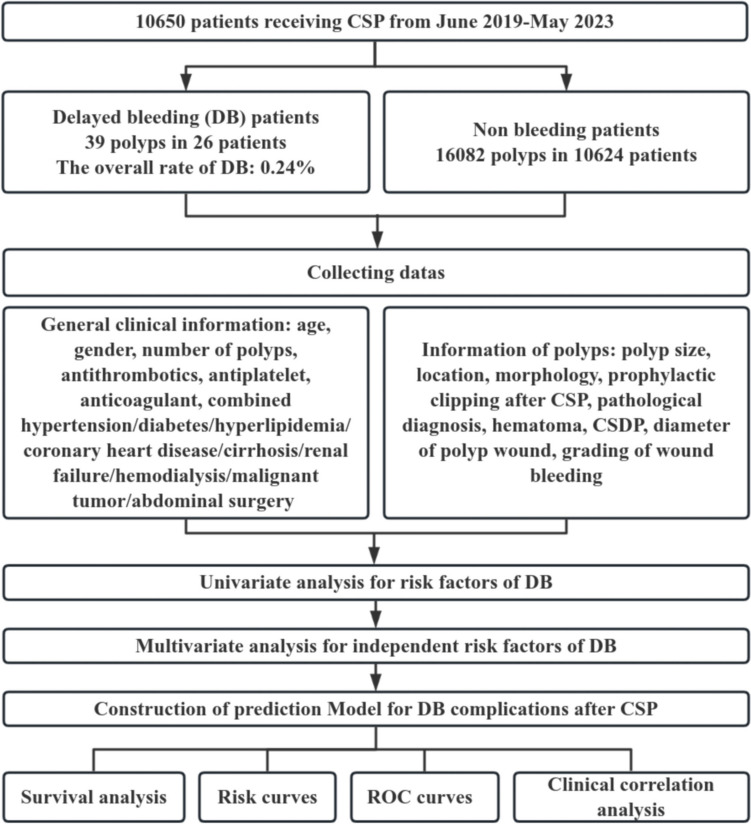
Experimental flowchart for risk factors of DB and the construction of the prognostic model of DB

The study adhered to STROBE reporting guideline and a diagram of the study scheme is shown in Fig. 1. This study is a retrospective cohort study conducted in 4 institutions of Guangdong Provincial Hospital of Chinese Medicine in Guangzhou, Guangdong, China. There were 10,650 patients underwent CSP for polyps in the colon from June 2019 to May 2023. We extracted the general clinical information and polyp-related information of patients with postoperative DB. The general information included age, gender, number of polyps, Boston bowel preparation scale scores (BBPS), history of hypertension (HTN), diabetes (DM), hyperlipidemia (HPL), coronary artery disease (CAD), renal failure (RF), liver cirrhosis (LC), malignant tumor (MT), and history of abdominal operation (AO), as well as the rate of antithrombotics (AT) use, antiplatelet (AP) use, and rate of anticoagulant (AC) use. Meanwhile, we collected the number of days when patients had postoperative DB.

Polyp-related information include polyp location, morphology (0-Ip, 0-Isp, 0-Is, 0-IIa), size, narrow-band imaging international colorectal endoscopic (NICE) classification, pathological diagnosis (adenomatous or non-adenomatous), hematoma, cold snare defect protrusion (CSDP), diameter of polyp wound, and grading of wound bleeding (grades 1 to 3). The grade of wound bleeding indicates the instantaneous wound bleeding after CSP. Grade 1 is almost no blood oozing, grade 2 is the blood oozing which is less than 50% of the wound surface, and grade 3 is the blood oozing which is more than 50% of the wound.

Meanwhile, 10624 non-DB patients with 16082 polyps who received CSP in Guangdong Provincial Hospital of Chinese Medicin were extracted as a control group. The general clinical information and polyp-related information of patients were also collected and the risk factors of DB after CSP were analyzed by a comparative study.

### Implementations of CSP

The patients were given a low fibre diet 2 days, and took 3L polyethylene glycol electrolyte solution and 30 ml simethicone for bowel preparation the night before endoscopy. Patients with chronic gastrointestinal conditions, such as inflammatory bowel disease (IBD), underwent bowel preparation utilizing evidence-based protocols [9]. The electronic endoscope systems used in this study included CV-290 (Olympus, Japan) and EPX-4450HD (Fujifilm, Japan). Colonoscopy was performed using a CF-H290I, GIF-XQ260 colonoscope (Olympus Corp, Tokyo, Japan) or EC-720R/M colonoscope (Fujifilm, Tokyo, Japan). The snare we used was Captivator II Single Use Snare (M00561220, Boston Scientific Corporation, Middlesex County, MA, USA). Polyp sizes were calculated based on the size of the snares and their maximum diameter. CSP is suitable for lesions < 10 mm that are diagnosed as benign diseases and can be completely resected en bloc. For the flat/depressed type and lesions suspected to be cancer, even if the lesions are ≤ 5 mm, CSP should be avoided [10, 11]. Polyps are retrieved through a suction port or gauze when they are resected. On the basis of histopathology, polyps were classified as adenomas or non-adenomas lesions.

### Screening of risk factors of DB

The comparison of characteristics between DB cases and non-DB cases was conducted using the Chi-square test. Univariate Cox analysis was performed to examine the risk factors using “survival” package. Significant results with *p*-values less than 0.05 were displayed in a forest plot. High-risk factors had a hazard ratio (HR) > 1 while low-risk factors had a HR < 1.

### Construction of the prediction model for DB after CSP for colorectal polyps

After acquiring the risk factors with a *p*-value of < 0.05, we used the “survival” package to develop a prediction model using a multivariate Cox regression analysis. The model was utilized to evaluate the connection of the DB complications after CSP and the risk factors. Patients were divided into high and low risk groups based on their median risk scores using the model. With the “survival” and “survminer” packages, a Kaplan Meier log-rank analysis was conducted to validate the model. A receiver operating characteristic (ROC) curve and risk plot were then produced using the “survival ROC” package and “pheatmap” package, respectively.

### Clinical correlation analysis

The correlation between the prediction model and clinical factors in patients underwent CSP was assessed using the “ggpubr” package. The relevance analysis of the risk factors was conducted in the R software using “corrplot” package. Correlation coefficients (cor) > 0 indicated positive correlation, while cor < 0 indicated negative correlation. The study sets the filtering criteria as | cor|> 0.5 and the correlation network was presented using the “igraph” and “reshape2” packages.

### Ethics statement

Before analysis, the patient records and information were anonymized and de-identified. The study was reviewed and approved by the Ethics Committee of Guangdong Provincial Hospital of Chinese Medicine (ethics no. ZE2023-248–01, date of approval: July 13, 2023).

### Statistical analysis

Data with normal distribution were recorded as mean values and standard deviation (SD), and the skewed distribution data are presented as median and interquartile range. The normality test and homogeneity of variance test were conducted on the measurement data. A *T*-test was performed for data that exhibit normal distribution and homogeneity of variance, while a non-parametric test was utilized for data that did not meet these assumptions. The chi-square test was used for analyzing categorical variables. All analyses were conducted using the Stata SE 15 software and R version 4.2.1. Statistical significance will be set at *p* < 0.05.

## Results

### Patients and lesions characteristics and the rate of DB

In this study, 26 DB cases with 39 polyps were analyzed, and the overall rate of DB was 0.24% (26/10650). Hemostasis was achieved by endoscopic clipping. Blood transfusions were not administered in any cases. DB usually manifest within a range of 0 to 5 days following CSP, with an average of 1.58 ± 1.47 days. The numbers of patients of bleeding day after CSP [*n* (%)] (0/1/2/3/4/5) are 7, 8, 5, 4, 0, 2 (26.92, 30.77, 19.23, 15.38, 0.00, 7.69). On the first day after CSP, DB is more likely to occur.

**Fig. 2:**
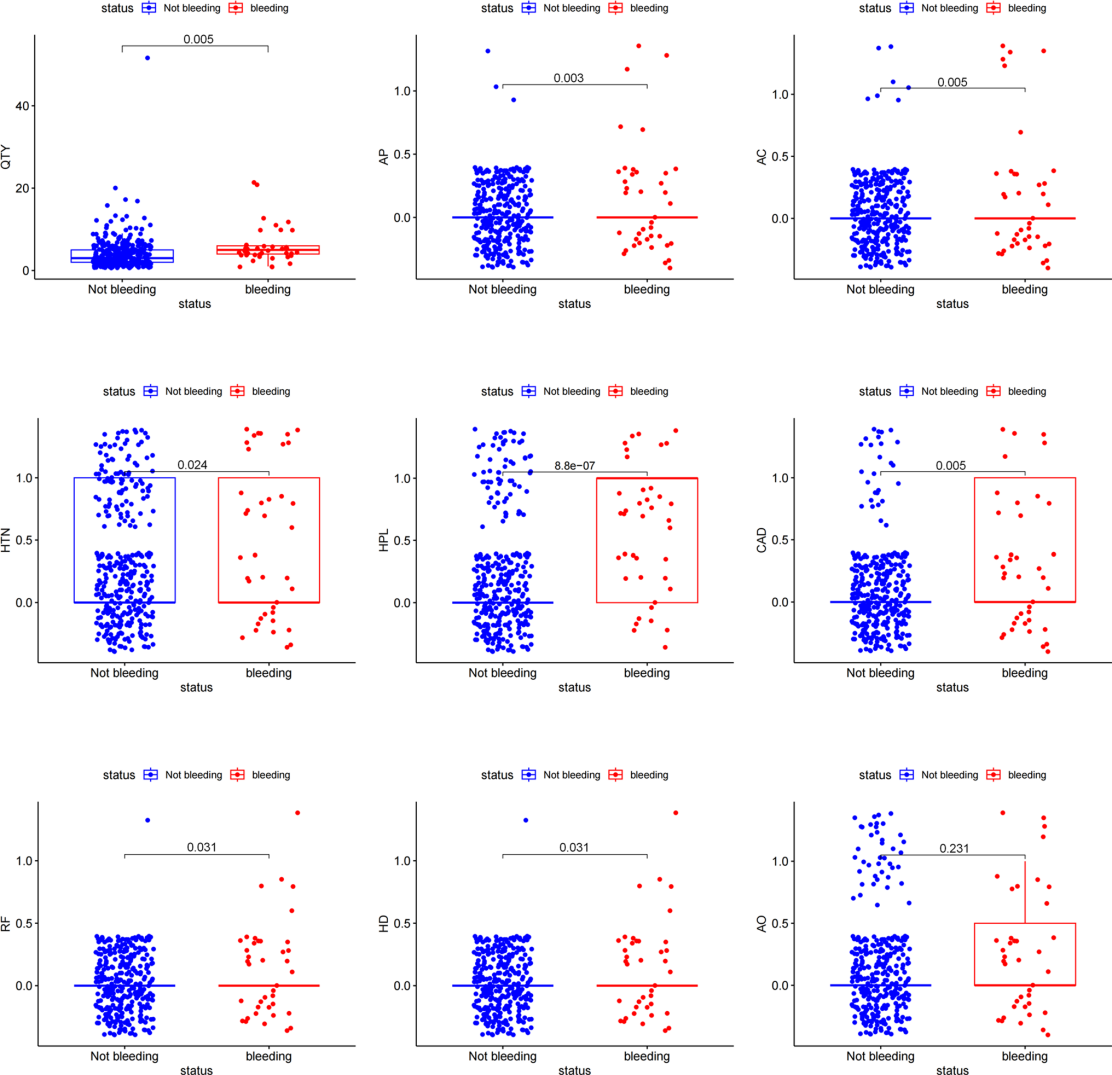
Comparison of characteristics between DB cases and non-DB cases with per-patient analysis

In the DB and non-DB groups, the median age was 61.00 (IQR, 49.75 to 66.25) years, 60.00 (IQR, 51.00 to 68.00) years (*p* = 0.842). The proportion of males was 57.69% and 58.72% (*p* = 1.000), respectively. In the DB group, quantity of polyps ≥ 3 (80.77% vs. 52.32%, *p* = 0.005), AP use (15.38% vs. 1.38%, *p* < 0.001), AC use (15.38% vs. 1.84%, *p* = 0.001), patients with history of HTN (50.00% vs. 27.55%, *p* = 0.015), HPL (57.69% vs. 19.28%, *p* < 0.001), CAD (26.92% vs. 7.36%, *p* = 0.002), and RF (7.69% vs. 0.46%, *p* = 0.007) were significantly higher than in the non-DB group. There were no significant differences in the antithrombotics use, patients with history of DM, LC, MT, and AO (Supplementary Table S1, Fig. 2).

**Fig. 3:**
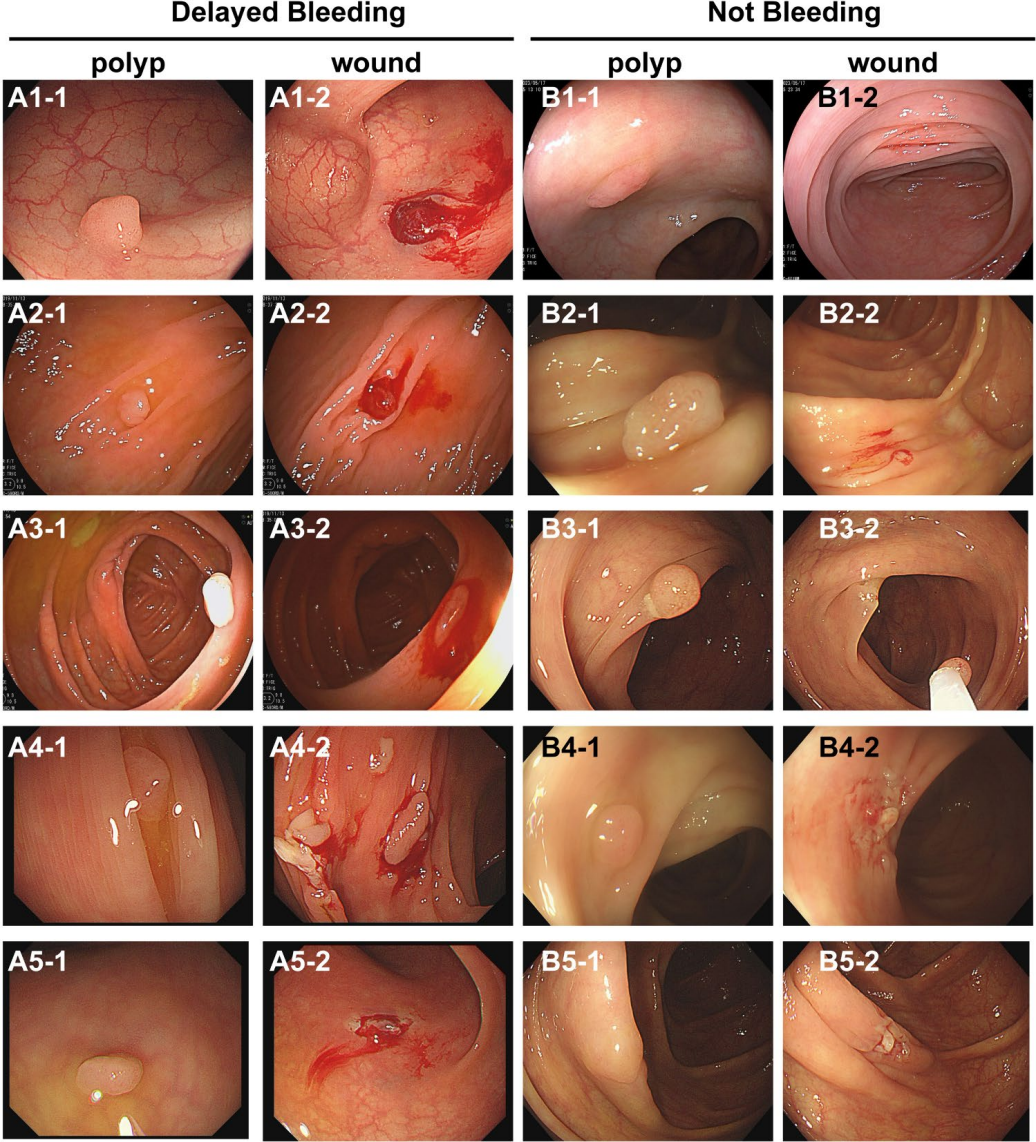
The polyps and wounds in DB patients (**A**) and non-DB patients (**B**). **A1** A 66-year-old female with polypoid lesion in sigmoid colon with a diameter of 7 mm, and the DB occurred 10 h after CSP. **A2** A 60-year-old male with polypoid lesion in hepatic flexure with a diameter of 9 mm, and the DB occurred 5 days after CSP. **A3** A 60-year-old male with polypoid lesion in ascending colon with a diameter of 9 mm, and the DB occurred 5 days after CSP. **A4** A 68-year-old male with polypoid lesion in ascending colon with a diameter of 8 mm, and the DB occurred 3 days after CSP. **A5** A 68-year-old male with polypoid lesion in sigmoid colon with a diameter of 6 mm, and the DB occurred 3 days after CSP. **B1** A 65-year-old female with polypoid lesion in transverse colon with a diameter of 6 mm. **B2** A 60-year-old female with polypoid lesion in ascending colon with a diameter of 7 mm. **B3** A 61-year-old male with polypoid lesion in transverse colon with a diameter of 6 mm. **B4** A 60-year-old female with polypoid lesion in descending colon with a diameter of 5 mm. **B5** A 49-year-old female with polypoid lesion in ascending colon with a diameter of 8 mm

**Fig. 4:**
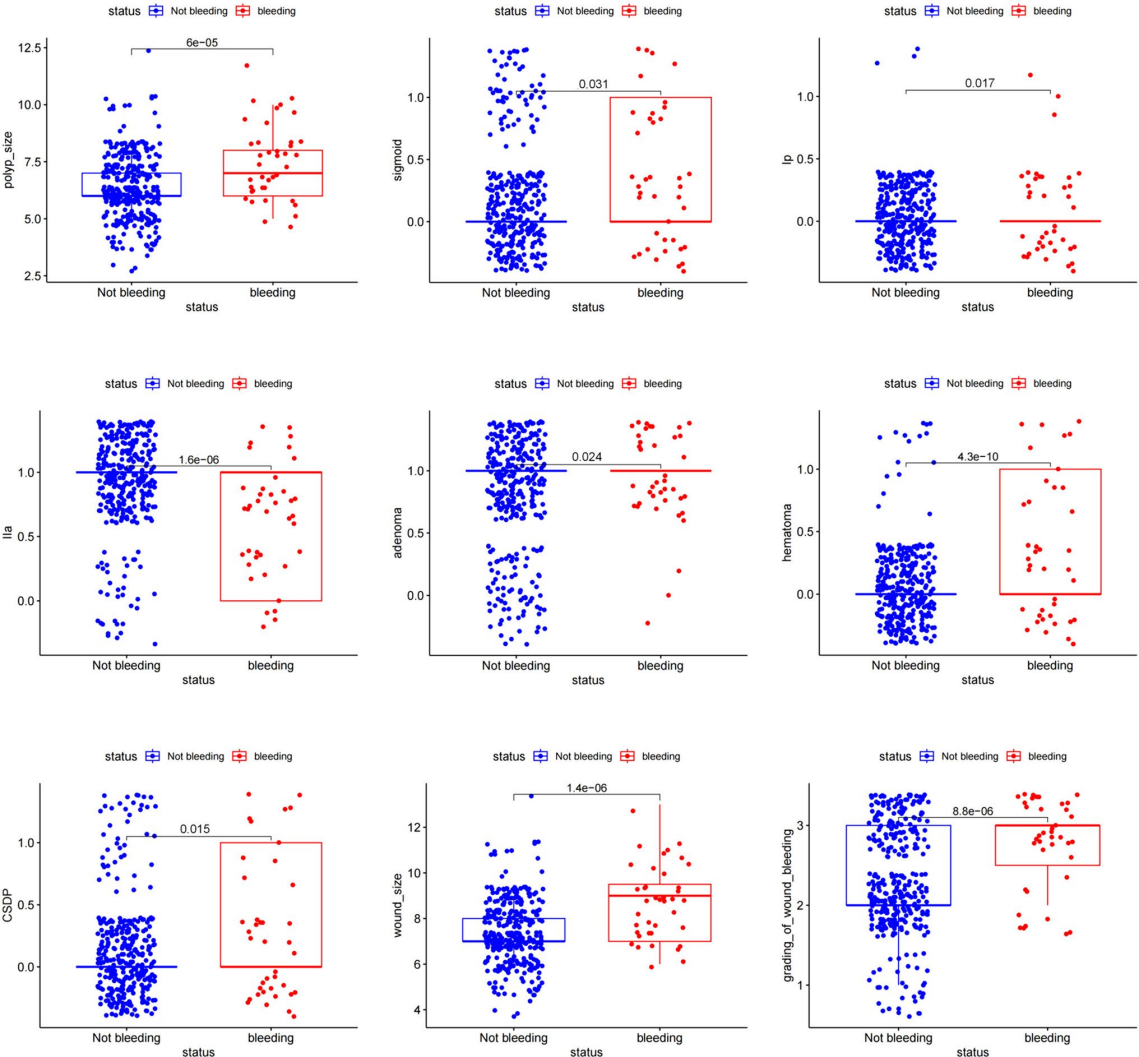
Comparison of characteristics between DB cases and non-DB cases with per-lesion analysis

The DB group also had significantly higher rates of sigmoid colon lesions (33.33% vs. 17.86%, *p* = 0.019), higher size of polyp (7 vs. 6, *p* < 0.001), morphology of type Is (28.21% vs. 7.59%, *p* < 0.001), morphology of type Ip (7.69% vs. 0.91%, *p* = 0.006), adenomatous (92.31% vs. 76.67%, *p* = 0.021), hematoma (33.33% vs. 4.85%, *p* < 0.001), CSDP (28.21% vs. 12.74%, *p* = 0.013), wound size (9 mm vs. 7 mm, *p* < 0.001), and grade 3 of wound bleeding (74.36% vs. 37.83%, *p* < 0.001) than the non-DB group (Fig. 3, Supplementary Figure S1). The rate of morphology of type IIa (61.54% vs. 89.40%, *p* < 0.001) was significantly lower than in the non-DB group (Supplementary Table S2, Fig. 4).

### The risk factors of DB

The univariate cox analysis of per-patient factors [hazard ratio (HR); 95% confdence interval (CI)] indicated history of HTN (2.244; 1.198–4.205, *p* = 0.012), HPL (4.965; 2.637–9.349, *p* < 0.001), CAD (4.384; 2.183–8.806, *p* < 0.001), RF (44.505; 17.405–113.797, *p* < 0.001), AT use (17.274; 4.163–71.671, *p* = 0.003), AP use (15.805; 6.181–40.410, *p* < 0.001), AC use (8.357; 3.502–19.945, *p* < 0.001), history of AO (2.428; 1.183–4.982, *p* = 0.016), HD (44.505; 17.405–113.797, *p* < 0.001), and quantity of polyps ≥ 3 (3.774; 1.476–9.650, *p* = 0.006) as independent risk factors for DB.

**Fig. 5:**
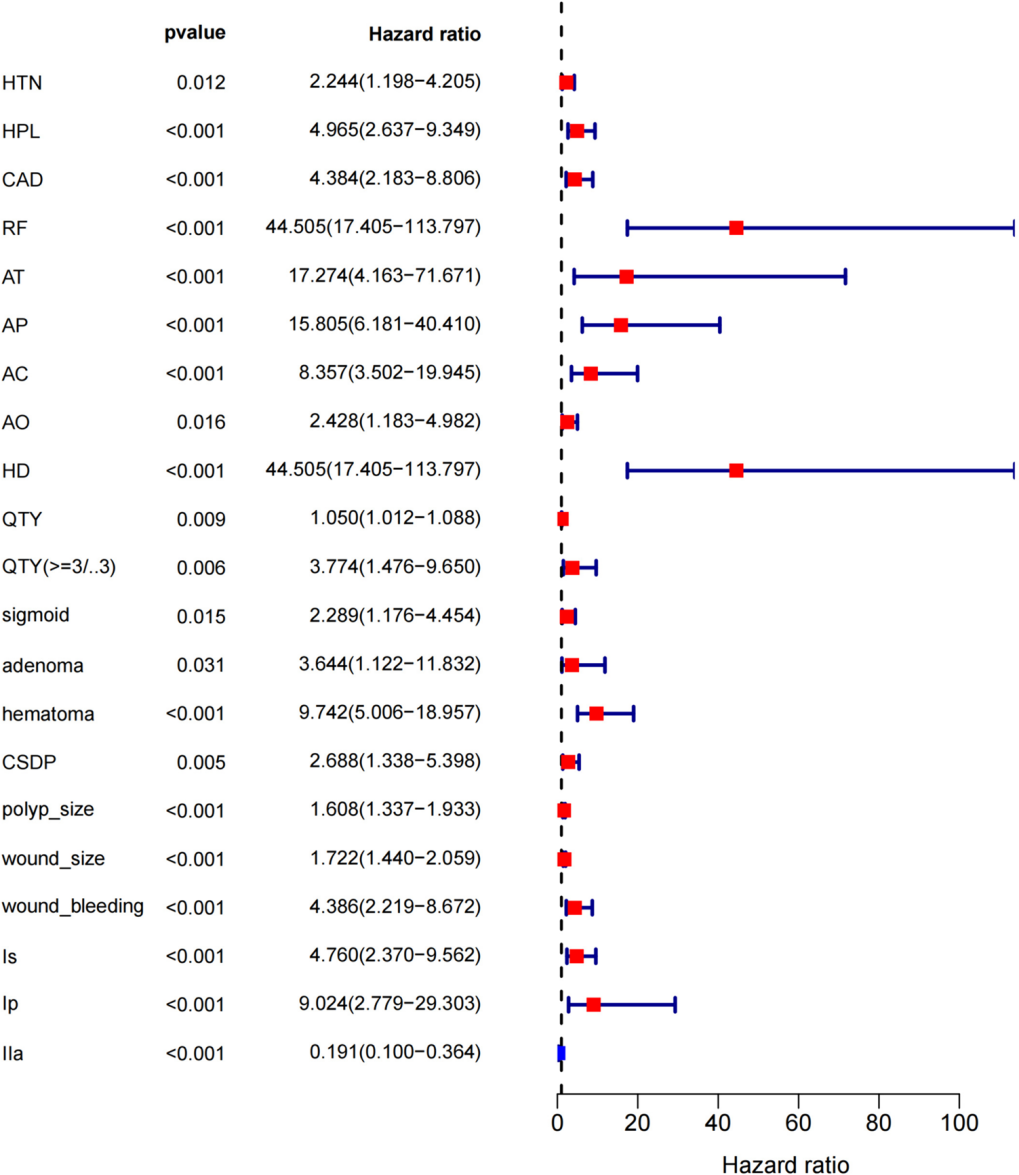
Forest plot of risk factors of DB after CSP for colorectal polyps

The univariate analysis of per-lesion factors showed sigmoid colon lesions (2.289; 1.176–4.454, *p* = 0.015), adenoma (3.644; 1.122–11.832, *p* = 0.031), hematoma (9.742; 5.006–18.957, *p* < 0.001), CSDP (2.688; 1.338–5.398, *p* = 0.005), polyp size (1.608; 1.337–1.933, *p* < 0.001), wound size (1.722; 1.440–2.059, *p* < 0.001), the grade of wound bleeding (4.386; 2.219–8.672, *p* < 0.001), Is (4.760; 2.370–9.562, *p* < 0.001), and Ip (9.024; 2.779–29.303, *p* < 0.001) as independent risk factors for DB (Supplementary Table S3, Fig. 5).

### Construction of the prediction model for DB after CSP for colorectal polyps

The multivariate Cox analysis of factors showed history of HPL (5.939; 2.627–13.43, *p* < 0.001), AT use (26.978, 5.177–140.576), AP use (97.950; 30.721–312.302, *p* < 0.001), AC use (14.493;4.855–43.261, *p* < 0.001), history of AO (5.340; 2.343–12.168, *p* < 0.001), sigmoid colon lesions (2.697; 1.234–5.894, *p* = 0.013), hematoma (6.855; 2.417–19.436, *p* < 0.001), wound size (1171.495; 408.703–3357.944, *p* < 0.001), the grade of wound bleeding (4.069; 1.568–10.565, *p* = 0.004), and Ip (28.422; 6.862–117.728, *p* = 0.005) as risk factors for DB (Supplementary Table S3).

**Fig. 6:**
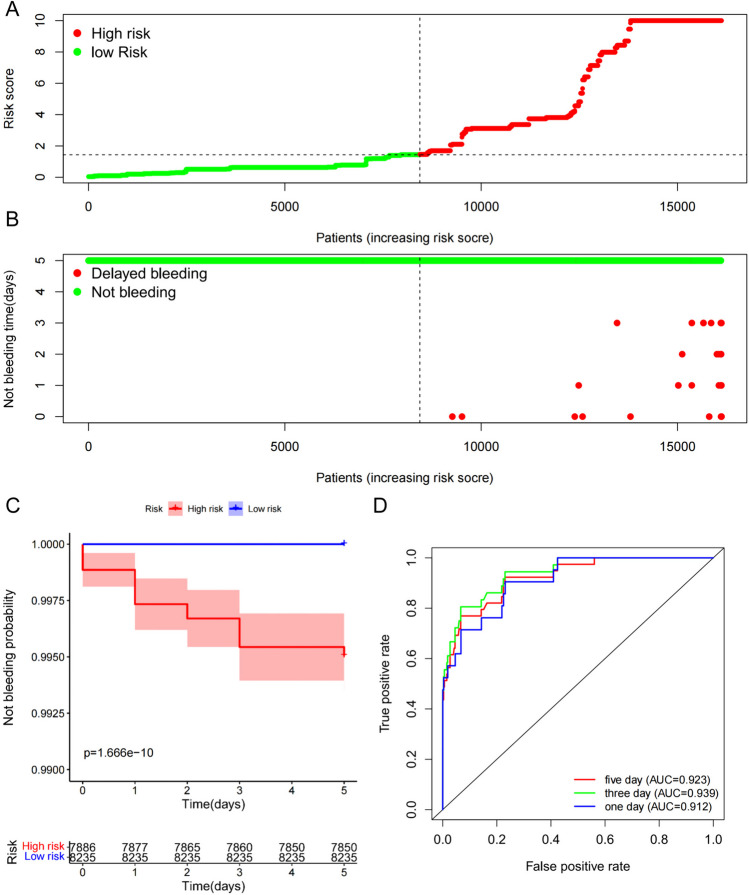
Construction of prediction model for DB complications after CSP. **A** The risk score distribution of the patients. **B** The status of whether DB and not bleeding time of each patient. **C** Survival analysis. **D** Time-dependent ROC curves of prediction model. The area under the ROC of 1-day, 3-day, and 5-day were 0.894, 0.944, 0.929, respectively, demonstrating the model’s good discriminative ability

The prediction model for DB after CSP for colorectal polyps was developed. Based on the numeration data variable of the factors and the coefficient, the formula was presented as: risk score = e^[ (HTN× −0.956) + (HPL×1.782) + (AT×3.295) + (AP×4.584) + (AC×2.674) + (AO×1.675) + (sigmoid×0.992) + (hematoma×1.925) + (CSDP× −1.582) + (polyp_size× −6.868) + (wound_size×7.066) + (the grade of wound bleeding×1.404) + (Ip×3.347)] −6.546^. In accordance with the median score of 1.450, the polyps were divided into high-risk group (*n* = 7886) and low-risk group (*n* = 8235) (Fig. 6A). A status overview was established (Fig. 6B). The Kaplan–Meier analysis confirmed the probability of DB after CSP would be greater for high-risk group compared to those in the low-risk group (Fig. 6C). For 1-, 3-, and 5-day overall bleeding, the AUCs of the ROC curves were 0.912, 0.939, and 0.923, respectively. The finding indicated that the prediction model exhibits favorable sensitivity and specificity (Fig. 6D).

### Clinical correlation analysis

We performed a correlation analysis between the prediction model and clinical factors in patients. It was shown that the rate of HPL, QTY, sigmoid colon, AP, AC, AO, hematoma, CSDP, and Ip type was positively related to the risk score (Supplementary Figure S2). The correlation heatmap showed the correlation of clinical factors (Supplementary Figure S3A). A correlation network composed of 5 nodes and 7 edges (Supplementary Figure S3B) was constructed to reveal the relationship among the clinical factors. The results shown that polyp size and wound size have positive correlations with hematoma, CSDP, and the grade of wound bleeding.

## Discussion

Colorectal polyps are common clinical disease, and some colorectal polyps can deteriorate into CRC. Endoscopic polypectomy is an important means to prevent CRC. It is reported that CSP had some advantages, such as short operation time, high complete resection rate, quicker mucosal defect repair [12] and low complication rate for colorectal polyps with a diameter < 10 mm [13, 14], and is being gradually accepted by endoscopist [15]. Others have reported that CSP can be also applied as a safe and effective method to remove colorectal polyps with large diameter (> 10 mm) [16, 17]. DB is a common complication after CSP of colorectal polyps, which directly affects the prognosis of patients. Therefore, it is important to explore risk factors affecting DB after CSP.

In our study, patients had pre-existing hypertension, hyperlipidemia, coronary artery disease, renal failure, hemodialysis, abdominal operation, antithrombotics use, antiplatelet use, anticoagulant use, quantity of polyps ≥ 3, sigmoid colon lesions, adenoma, hematoma, CSDP, polyp size, wound size, and the grade of wound bleeding, polyp morphology Is and Ip which were independent risk factors for DB by univariate regression analysis. Then, we constructed a prediction model after a multivariate Cox regression analysis. By using the model, the risk score was calculated. Furthermore, the Kaplan–Meier analysis, risk analysis, ROC curve, and the clinical correlation analysis were all conducted to demonstrate the accuracy of the model.

Patients with hypertension and coronary heart disease experience decreased arterial elasticity and hardened arterial blood vessels [18, 19]. The vasoconstriction force of polyp stumps is reduced after CSP; meanwhile, the blood flow of patients with hypertension is in a state of high pressure, which will greatly increase the risk of DB after CSP. In the state of hyperlipidemia in the body, the ability of wound fibroblasts to synthesize collagen will be reduced and directly affects the wound healing [20], which is an inducing factor leading to delayed postoperative hemorrhage.

Patients with renal failure have an increased risk of vascular atherosclerosis and thrombosis, and decreased coagulation function, which greatly increases the risk of bleeding [21]. Studies have shown that patients with renal failure and dialysis are more likely to experience symptoms of gastrointestinal bleeding [22], and hemodialysis is a risk factor of post-polypectomy bleeding [23]. If the patient has a history of abdominal operation, abdominal visceral adhesions may be occurred, increasing the difficulty of CSP for the endoscopist. Taking antithrombotics, antiplatelet or anticoagulant can affect the coagulation function of the patient, thus increasing the risk of developing DB [24].

The present study shows the quantity of polyps was positively correlated with DB. In parallel, CSP of polyps located in the sigmoid colon is prone to DB, which may be related to its abundant vascular mucosa. The diameter of polyps and the wound size were significantly correlated with the occurrence of DB after CSP. The larger the polyps, the thicker the blood vessels, and the richer the blood supply of the polyps, the more difficult the operation of CSP, and the greater the risk of bleeding. Besides, the larger the polyp, the wider the surgical resection site, which increases the damage to the mucosa and is more prone to bleeding.

Compared with hyperplastic polyps, adenomatous polyps tend to have more abundant blood vessels and have more vascular stumps after CSP, thus have a higher risk of DB. Adenomatous polyps include tubular adenomas, villous adenomas, and tubulovillous adenomas [25]. Tubular adenomas are usually pedunculated with a nodular surface [26]. Villous adenoma has a wide base and generally has no pedicle, but contains many blood vessels, so it is easy to bleed after CSP. Tubulovillous adenomas have the common characteristics of tubular adenoma and villous adenoma [27]. It can be seen that the increased incidence of DB after CSP for adenomatous polyps may be related to the pedunculated polyps and abundant blood vessels. It has been suggested that the risk of bleeding after CSP is higher for polyps with macroscopic protruding appearance [28]. During endoscopic polypectomy, incomplete removal of pedunculated polyps is likely to occur. It is more likely to increase the risk of postoperative delayed bleeding that the residual pedicle is too long and involves more blood vessels.

CSP may have the incidence of incomplete mucosal layer resection. As the shape of the tip of the snare is fixed, the tissues will be mechanically torn after the snare is tightened, and some wounds will form CSDP during the process of CSP [29]. CSDP is mainly composed of submucosa and some residual muscularis mucosa [30]. When resecting mucosal lesions, the vascular stump is thicker or more, the oozing blood can quickly fill the submucosa of the wound to form a hematoma, resulting in bleeding that cannot be stopped within a short time after CSP, increasing the risk of DB. In addition, when the mucosal layer of the wound is completely resected, no CSDP is formed on the wound [31], but the grade of wound bleeding is high, and the wound quickly oozes with blood or hemorrhages hematoma immediately after the polyp is removed, it also indicates that there are many blood vessels at the stump or the blood vessels are relatively thick, so it is necessary to pay attention to additional endoscopic hemostasis operations.

Our study provides a new view of the risk factors affecting DB after CSP for polyps in the colon. Meanwhile, a prediction model was constructed, suggesting that the risk factors affecting DB should be fully considered. For the high-risk patients of DB, active measures such as endoscopic hemostasis should be taken early to reduce the occurrence of DB and improve safety and efficiency of the treatment. Although these results are promising, some limitations of this study should be taken into consideration. This study was retrospective, so it had limitations common to retrospective studies. Additional factors, such as the utilization of water-assisted colonoscopy technology (both water-immersion and water-exchange), were not considered in this study and require further exploration. The number of positive cases was limited and the analyses were based on the data collected in four hospitals in Guangzhou, limiting its generalizability. Thus, the results may be highly valid internally, but less valid externally. Therefore, a larger prospective multicenter study is needed in the future to confirm the findings of this study.

## Supplementary information

Below is the link to the electronic supplementary material.

Supplementary file1 (TIF 24.7 MB)

Supplementary file2 (TIF 1.43 MB)

Supplementary file3 (TIF 2.35 MB)

Supplementary file4 (DOCX 24.7 KB)

Supplementary file5 (DOC 100 KB)

## Data Availability

All data generated or analyzed during this study are included in this article.

## Abbreviations

CI: Confidence intervals
CSP: Cold snare polypectomy
CSDP: Cold snare defect protrusion
CRC: Colorectal cancer
DB: Delayed bleeding
HR: Hazard ratios
BBPS: Boston bowel preparation scale scores
HTN: History of hypertension
DM: History of diabetes
HPL: History of hyperlipidemia
CAD: History of coronary artery disease
RF: Renal failure
LC: Liver cirrhosis
AT: Use of antithrombotics
AP: Use of antiplatelet
AC: Use of anticoagulant
AT_AP_AC: Use of antithrombotics or antiplatelet or anticoagulant
MT: Malignant tumor
AO: History of abdominal operation
HD: History of hemodialysis
HR: Hazard ratio
NICE: Narrow-band imaging international colorectal endoscopic
ROC: Receiver operating characteristic
Cor: Correlation coefficients
SD: Standard deviation
QTY: Quantity of polyps
