# Supplementary figures and images for: Risk factors and prediction model for delayed bleeding after cold snare polypectomy: a retrospective study
Source: Int J Colorectal Dis. 2024 Jul 22;39(1):113. doi: 10.1007/s00384-024-04687-8 (PMC11263232; doi:10.1007/s00384-024-04687-8)

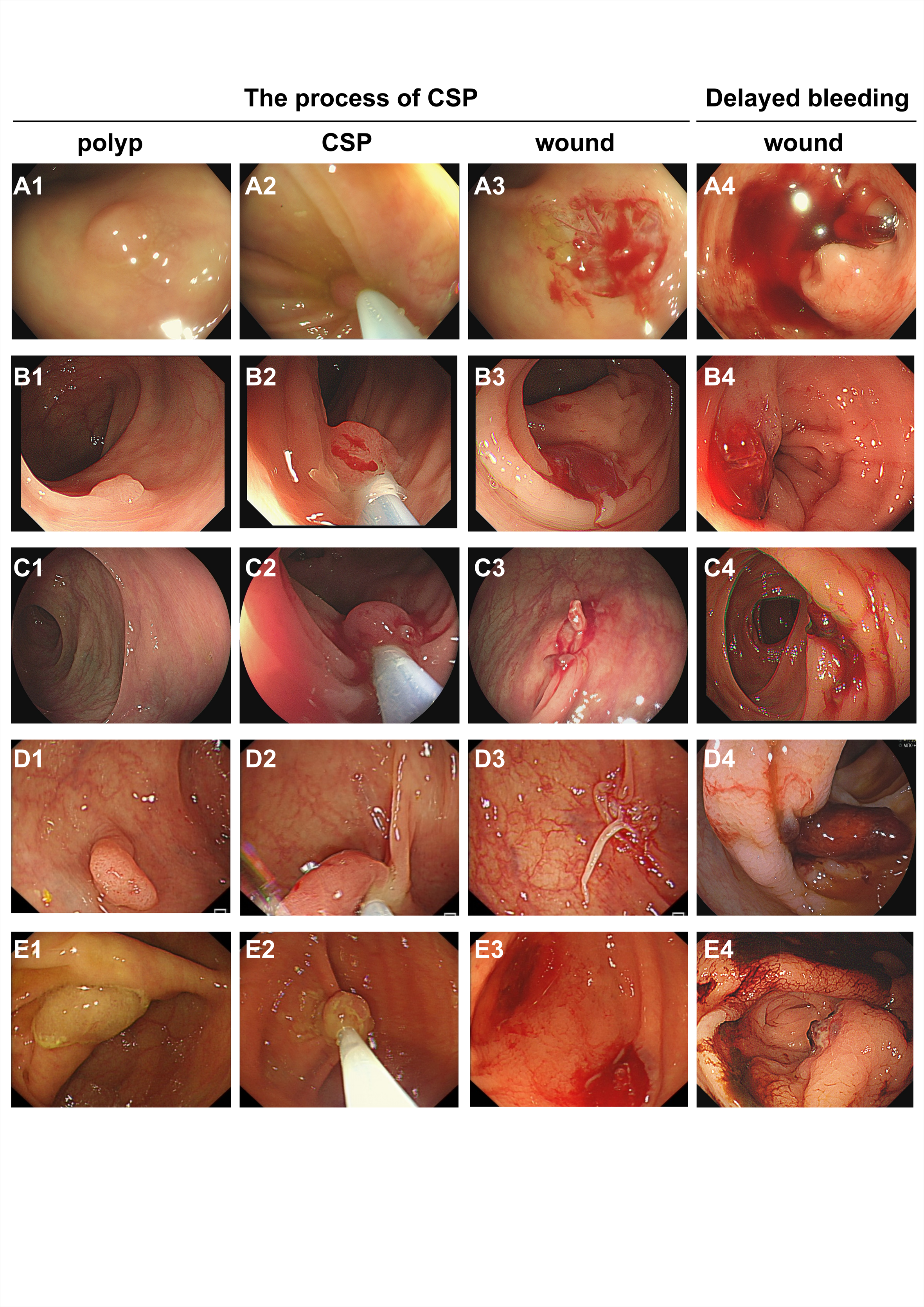

Supplement: Supplementary file 1 — Supplementary file1 (TIF 24.7 MB) [file 384_2024_4687_MOESM1_ESM.tif]

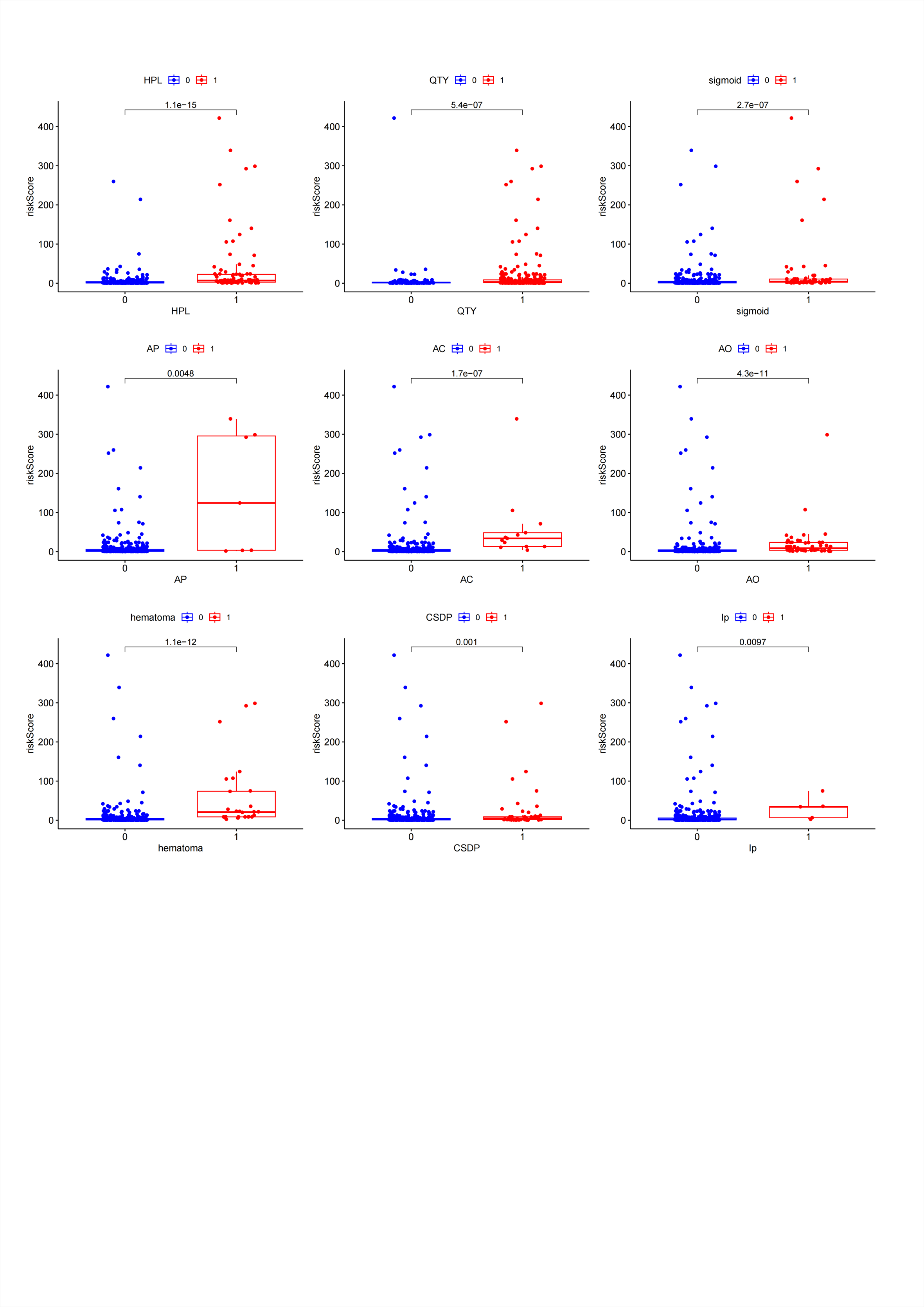

Supplement: Supplementary file 2 — Supplementary file2 (TIF 1.43 MB) [file 384_2024_4687_MOESM2_ESM.tif]

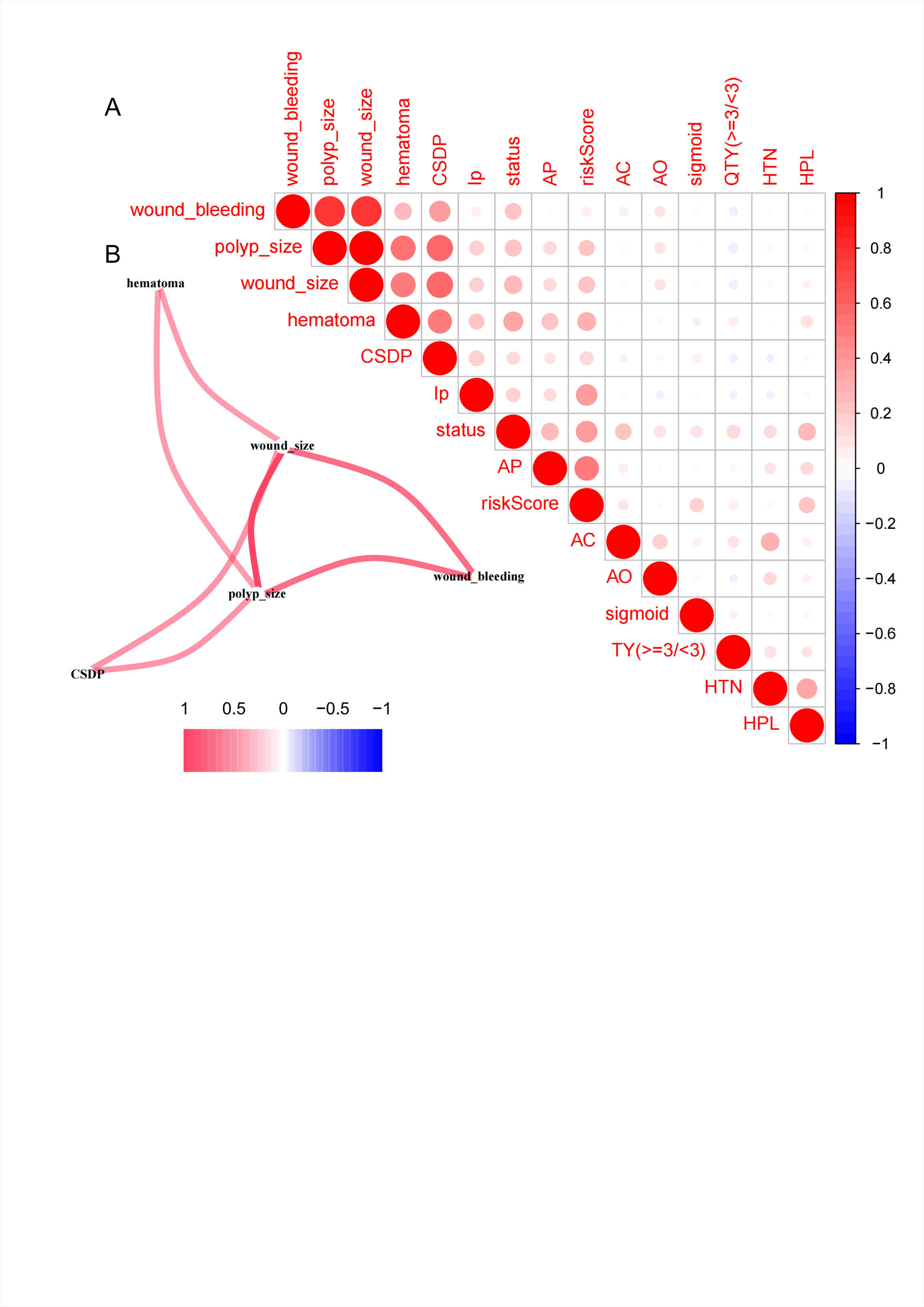

Supplement: Supplementary file 3 — Supplementary file3 (TIF 2.35 MB) [file 384_2024_4687_MOESM3_ESM.tif]
