# Supplementary material for: Risk factors and prediction model for delayed bleeding after cold snare polypectomy: a retrospective study
Source: Int J Colorectal Dis. 2024 Jul 22;39(1):113. doi: 10.1007/s00384-024-04687-8 (PMC11263232; doi:10.1007/s00384-024-04687-8)
Supplement: Supplementary file 4 — Supplementary file4 (DOCX 24.7 KB) [file 384_2024_4687_MOESM4_ESM.docx]

| **Supplementary Table S1** Characteristics of DB cases and non-DB cases: per-patient analysis | | | | |
| --- | --- | --- | --- | --- |
|  | | DB (n=26) | Non-DB (n=10624) | P value |
| Age, years, M(P25,P75) | 61.00(49.75,66.25) | | 60.00(51.00,68.00) | 0.827 |
| Sex, n (%)(male/female) | 15/11 (57.69/42.31) | | 6238/4386 (58.72/41.28) | 1.000 |
| Quantity of polyps，M(P25,P75) | 4 (3, 6) | | 3 (2, 5) | 0.003 |
| Quantity of polyps[n (%)]（≥3 /＜3） | 21/5 (80.77/19.23) | | 5558/5066 (52.32/47.68) | 0.005 |
| Medications, antithrombotics [n(%)] | 1/25 (3.85/96.15) | | 49/10575 (0.46/99.54) | 0.115 |
| Medications, antiplatelet [n (%)] | 4/22 (15.38/84.62) | | 147/10477 (1.38/98.62) | <0.001 |
| Medications, anticoagulant [n (%)] | 4/22 (15.38/84.62) | | 195/10429 (1.84/98.16) | 0.001 |
| Comorbidity, hypertension[n (%)] | 13/13 (50.00/50.00) | | 2927/7697 (27.55/72.45) | 0.015 |
| Comorbidity, diabetes[n(%)] | 4/22 (15.38/84.62) | | 1515/9109 (14.26/85.74) | 0.781 |
| Comorbidity, hyperlipidemia[n (%)] | 15/11 (57.69/42.31) | | 2048/8576 (19.28/80.72) | <0.001 |
| Comorbidity, coronary artery disease[n(%)] | | 7/19 (26.92/73.08) | 782/9842 (7.36/92.67) | 0.002 |
| Comorbidity, liver cirrhosis[n(%)] | | 1/25 (3.85/96.15) | 146/10478 (1.37/98.63) | 0.304 |
| Comorbidity, renal failure[n (%)] | | 2/24 (7.69/92.31) | 49/10575 (0.46/99.54) | 0.007 |
| Comorbidity, malignant tumor | | 4/22 (15.38/84.62) | 1122/9502 (10.56/89.44) | 0.348 |
| Comorbidity, abdominal operation | | 6/20 (23.08/76.92) | 1413/9211 (13.30/86.70) | 0.147 |
| BBPS score | | 6.00 (5.75, 8.00) | 7.00 (6.00, 8.00) | 0.116 |

| **Supplementary Table S2** Comparison of characteristics between DB cases and non-DB cases: per-lesion analysis | | | | |
| --- | --- | --- | --- | --- |
|  | DB (n=39) | Non-DB (n=16082) | | P value |
| Mean polyp size, M(P25,P75)(mm) | 7.00(6.00,8.00) | 6.00(6.00,7.00) | <0.001 | |
| Location [n (%)](cecum/ascending colon/hepatic flexure/transverse colon/descending colon/sigmoid colon/rectum) | 2/5/1/9/6/13/3(5.13/12.82/2.56/23.08/15.38/33.33/7.69) | 682/3215/681/4581/2922/2871/1120(4.24/20.00/4.24/28.50/18.18/17.86/6.98) | 0.31 | |
| Location[n(%)](sigmoid colon/non-sigmoid colon) | 13/26(33.33/66.67) | 2871/13211(17.86/82.14) | 0.019 | |
| NICE classification (NICE type 1/ NICE type 2) | 3/36(7.69/92.31) | 3749/12333(23.33/76.67) | 0.021 | |
| Morphology [n (%)] (Is/Isp/Ip/IIa) | 11/1/3/24(28.21/2.56/7.69/61.54) | 1220/342/146/14374(7.59/2.13/0.91/89.37) | <0.001 | |
| Morphology [n (%)] (Is/Non-Is) | 11/28(28.21/71.79) | 1220/14862(7.59/92.41) | <0.001 | |
| Morphology [n (%)] (Ip/Non-Ip) | 3/36(7.69/92.31) | 146/15936(0.91/99.09) | 0.006 | |
| Morphology [n (%)] (IIa/Non-IIa) | 24/15(61.54/38.46) | 14374/1708(89.40/10.60) | <0.001 | |
| Pathological diagnosis[n(%)] (adenomatous/non-adenomatous) | 36/3(92.31/7.69) | 12333/3749(76.67/23.33) | 0.021 | |
| hematoma[n(%)] | 13/26(33.33/66.67) | 780/15302(4.85/95.15) | <0.001 | |
| CSDP[n(%)] | 11/28(28.21/71.79) | 2049/14033(12.74/87.266) | 0.013 | |
| wound size, M(P25,P75)(mm) | 9.00(7.00,10.00) | 7.00(6.75,8.00) | <0.001 | |
| grading of wound bleeding[n(%)](grade 1/2/3) | 0/10/29(0.00/25.64/74.36) | 1661/8337/6084(10.33/51.84/37.83) | <0.001 | |

| **Supplementary Table S3** Univariate and multivariate analyses for DB | | | | | | |
| --- | --- | --- | --- | --- | --- | --- |
| Parameter | Univariate analysis | | | Multivariate analysis | | |
|  | HR | 95% CI | P | HR | 95% CI | P |
| **Risk factors: per-patient analysis** | | | | | | |
| history of hypertension | 2.244 | 1.198- 4.205 | 0.012 | 0.384 | 0.152- 0.97 | 0.043 |
| history of hyperlipidemia | 4.965 | 2.637- 9.349 | <0.001 | 5.939 | 2.627- 13.43 | <0.001 |
| medications antithrombotics | 17.274 | 4.163- 71.671 | <0.001 | 26.978 | 5.177- 140.576 | <0.001 |
| medications antiplatelet | 15.805 | 6.181- 40.41 | <0.001 | 97.950 | 30.721- 312.302 | <0.001 |
| medications anticoagulant | 8.357 | 3.502- 19.945 | <0.001 | 14.493 | 4.855- 43.261 | <0.001 |
| history of abdominal operation | 2.428 | 1.183- 4.982 | 0.016 | 5.340 | 2.343- 12.168 | <0.001 |
| **Risk factors: per-lesion analysis** | | | | | | |
| Location(sigmoid colon/non-sigmoid colon) | 2.289 | 1.176- 4.454 | 0.015 | 2.697 | 1.234- 5.894 | 0.013 |
| hematoma | 9.742 | 5.006- 18.957 | <0.001 | 6.855 | 2.417- 19.436 | <0.001 |
| CSDP | 2.688 | 1.338- 5.398 | 0.005 | 0.206 | 0.068- 0.621 | 0.005 |
| polyp size | 1.608 | 1.337- 1.933 | <0.001 | 0.001 | 0- 0.003 | <0.001 |
| wound size | 1.722 | 1.44- 2.059 | <0.001 | 1171.495 | 408.703- 3357.944 | <0.001 |
| grading of wound bleeding | 4.386 | 2.219- 8.672 | <0.001 | 4.069 | 1.568- 10.565 | 0.004 |
| Morphology(Ip/non-Ip) | 9.024 | 2.779- 29.303 | <0.001 | 28.422 | 6.862- 117.728 | <0.001 |
